# Natural Progression of Cranial Shape Following Helmet Therapy for Deformational Plagiocephaly

**DOI:** 10.3390/jcm14020357

**Published:** 2025-01-09

**Authors:** Risa Kato, Nobuhiko Nagano, Mari Sasano, Koichiro Sumi, Ichiro Morioka

**Affiliations:** 1 Department of Pediatrics and Child Health, Nihon University School of Medicine, Tokyo 173-8610, Japan; kato.risa@nihon-u.ac.jp (R.K.); morioka.ichiro@nihon-u.ac.jp (I.M.); 2 Department of Neurological Surgery, Kanagawa Children’s Medical Center, Yokohama 232-8555, Japan; sasano.mari@nihon-u.ac.jp; 3 Department of Neurological Surgery, Nihon University School of Medicine, Tokyo 173-8610, Japan; sumi.koichiro@nihon-u.ac.jp

**Keywords:** cranial shape, deformational plagiocephaly, natural progression, severity, helmet therapy

## Abstract

**Objectives:** This study aimed to examine the natural progression of the cranial shape from the end of helmet therapy for deformational plagiocephaly to 1 year of age. **Methods:** This study included infants with moderate to severe deformational plagiocephaly who began treatment at our department between December 2022 and July 2023. The cranial shape was assessed using a 3D scanner (VECTRA^®^H2) at the start of treatment, end of treatment, and 12 months of age. Changes in the Cranial Vault Asymmetry Index (ΔCVAI), an indicator of cranial deformity, and the severity of deformity (normal, mild, moderate, severe, very severe) were assessed between the end of treatment and 12 months. **Results:** The study included 32 infants: 30 were full-term and 2 were preterm (gestational ages: 34 and 36 weeks). The median age at the start of treatment was 4 months (range: 2–7 months), with a mean CVAI of 10.5 ± 2.2%. At the end of treatment, the median age was 9 months (range: 5–11 months), with a mean CVAI of 4.2 ± 1.8%. The ΔCVAI from the end of treatment to 12 months of age was 0.3 ± 0.8%. Regarding severity, an improvement was observed in one infant (3%) (from moderate to mild), no change in 28 infants (88%) (23 classified as normal, three as mild, and two as moderate), and worsening in three infants (9%) (one from mild to moderate and two from normal to mild). **Conclusions:** The severity of cranial deformity showed minimal worsening during the natural progression from the end of helmet therapy to 1 year of age.

## 1. Introduction

Deformational plagiocephaly (DP) is a head deformity caused by external pressure, such as the confined space in the mother’s uterus, the method of delivery, and the mild pressure exerted by the supine sleeping position [1].

In 1992, the American Academy of Pediatrics recommended placing healthy babies on their backs or sides to sleep to reduce the risk of sudden infant death syndrome (SIDS). As a result of the Back to Sleep campaign, which aimed to prevent SIDS, cases of DP have significantly increased [2,3].

In Japan, the number of SIDS-related deaths decreased from 526 in 1995 to 47 in 2022 [4]. However, it is believed that the incidence of DP has risen, as observed in other countries [5,6,7,8]. Additionally, Japan has a cultural preference for placing babies on their backs to sleep. Our hospital has reported a growing number of parents expressing concerns about their infants’ head deformities, even during the COVID-19 pandemic [9].

Recently, due to the influence of Western practices, helmet therapy for DP has become increasingly available in many facilities across Japan. As a result, head deformities and their treatment are receiving more attention [6,8,10,11,12,13,14]. When inquiring about treatment for head shape, many caregivers ask whether the deformity recurs after helmet therapy. The head circumference grows significantly during the first 6 to 12 months of life [15,16,17,18]. Therefore, it is recommended to start helmet therapy during the early infant stage, ideally before 6 months of age, when the skull remains soft [8,19,20,21,22,23,24,25]. Studies have shown that starting helmet therapy by 8 months and wearing it for over 15 h a day can be effective [26]. While the degree of improvement decreases with age, treatment can still be effective for infants aged 12 to 18 months [27,28]. Given that the head circumference grows rapidly and the bones fuse naturally during infancy, the cranial shape may change even after helmet therapy is completed. However, there is limited research on the rate of cranial asymmetry correction [29], and few studies have examined how the cranial shape evolves after helmet therapy [30].

In this study, we aimed to clarify the natural course of the cranial shape from the end of helmet therapy for DP to the age of 1 year, focusing on infants with moderate to severe DP who received helmet therapy during infancy.

## 2. Materials and Methods

### 2.1. Participants and Study Methods

This study was a prospective cohort study conducted between December 2022 and July 2023, focusing on infants with moderate to severe DP who visited Nihon University Itabashi Hospital and began treatment at our department.

Initially, we collected the clinical characteristics of the participants, including the gender, gestational age, birth height, birth weight, head circumference, chest circumference, mode of delivery, intrauterine position, and twin status. We also gathered maternal clinical data, such as the age at delivery and parity (primiparous or multiparous).

Subsequently, we evaluated the infants’ cranial shape using a three-dimensional (3D) scanner at the start and end of helmet therapy, as well as at 1 year of age. We assessed the Cranial Vault Asymmetry Index (CVAI), an indicator of cranial deformity, along with the severity of the condition (normal, mild, moderate, severe, very severe) at each scan.

We conducted two studies using the above variables and severity as key factors.

Study 1: We assessed the value of the CVAI at the start of helmet therapy (T1), at the end of helmet therapy (T2), and at the chronological age of 1 year (T3). Additionally, we calculated the change rate of the CVAI (ΔCVAI) between each time point. We also examined whether significant differences existed between the CVAI measurements at each point using Friedman’s test. Statistical analysis was performed using JMP (version 17.2.0, SAS Institute Inc., Cary, NC, USA).

Study 2: We analyzed changes in the severity of cranial deformity between T1 and T2, as well as between T2 and T3.

The results regarding participant characteristics are presented as numbers (percentages) and medians (min–max). The ages at T1, T2, and T3 are reported as medians (min–max), while the CVAI values at each time point are expressed as means ± standard deviations.

### 2.2. Data Acquisition Using the 3D Scanner

The cranial shape of each participant was scanned using a 3D scanner, the VECTRA H2 (Canfield Scientific, NJ, USA). The CVAI was determined using a two-dimensional evaluation based on the 3D images.

To do this, we established a basic cross-section (Level 0) passing through the sellion and the left and right tragions, as outlined in previous studies [10,31]. The midpoint of both tragions was defined as the origin. Once these landmarks and the basic plane were set, we defined the Y-axis as the line passing through the sellion and the origin. The X-axis was defined as the line perpendicular to the Y-axis that crosses the origin on the basic cross-section. The Z-axis was defined as the line perpendicular to the basic plane that intersects the origin.

**Figure 1:**
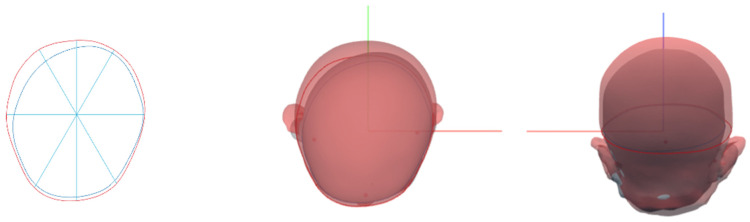
The appearance of the 3D images. The figure on the left shows a two-dimensional planar view (Level 3) derived from the 3D image. The figures in the center and on the right are 3D images of the infant’s head, created using a 3D scanner. This figure overlays the results of two scans of the same infant. By using different colors, the changes in the cranial shape over time are shown.

We then constructed 10 equidistant, parallel cross-sections through the upper part of the skull, with Level 10 being the highest. The third cross-section (Level 3) was selected to calculate the variable (Figure 1).

Cranial asymmetry (CA) was defined as the difference between the longer and shorter diagonals drawn at a 30° angle from the Y-axis. The CVAI was calculated as the quotient of CA divided by the shorter diagonal [32,33].

### 2.3. Severity of Cranial Deformity

We defined the severity of cranial deformity as follows:

CVAI < 5%: normal;
CVAI 5–6%: mild;
CVAI 7–9%: moderate;
CVAI 10–13%: severe;
CVAI ≥ 14%: very severe.

According to previous studies, the international diagnostic criterion for the CVAI is defined as >3.5% [32,34,35]. The recent Japanese literature often considers a CVAI of less than 5% as normal for cranial deformity, due to the higher prevalence of positional plagiocephaly and the cultural practice of back sleeping [10]. In this study, we set 5% as the threshold between normal and mild cranial deformity.

### 2.4. Cranial Remolding Helmet

We used a single helmet, the Baby Band 2 (Medical Device Approval No.: 30400BZX00252000), developed by Berry Inc. and introduced in November 2022. The therapeutic efficacy and safety of this device have been demonstrated in the previous literature, with the details provided in the same report [10].

The helmet was worn for as long as possible, except during bathing, care time, or when the child was unwell. As a general guideline, we recommended wearing it for more than 20 h a day. After starting helmet therapy, patients visited our hospital approximately once per month to ensure that they experienced no side effects from the helmets. We also verified that sufficient room remained for cranial growth and resolved any emerging issues that may have occurred, such as skin troubles.

The treatment was mainly concluded when the space inside the helmet became extremely small and tight or when the guardians were satisfied with the patient’s cranial shape.

## 3. Results

A total of 103 infants visited our hospital and started helmet therapy during the study period. Of these, 16 infants with normal (CVAI < 5%: normal) to mild (CVAI 5–6%: mild) plagiocephaly were excluded. An additional 55 infants who did not visit our hospital by the chronological age of 1 year were also excluded. The final study population consisted of 32 participants, 30 of whom were term infants and 2 were preterm infants (gestational ages of 34 and 36 weeks). Table 1 provides the clinical characteristics of the infants, while Table 2 presents the clinical characteristics of the mothers.

### 3.1. Study 1: Changes in CVAI

**Figure 2:**
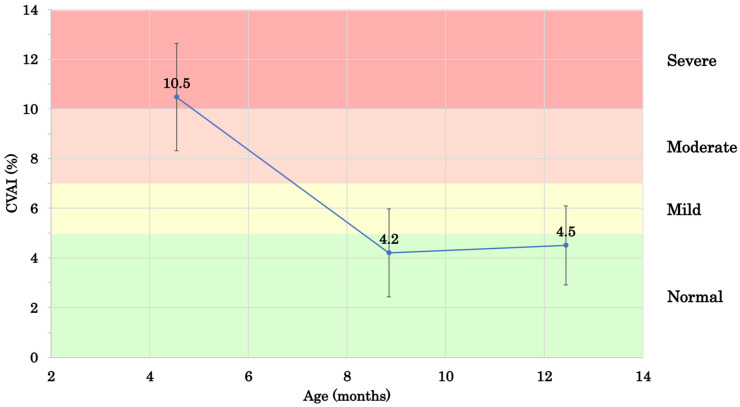
Changes in mean CVAI (mean ± standard deviation) from the start of helmet therapy to the chronological age of 1 year. CVAI, Cranial Vault Asymmetry Index.

**Figure 3:**
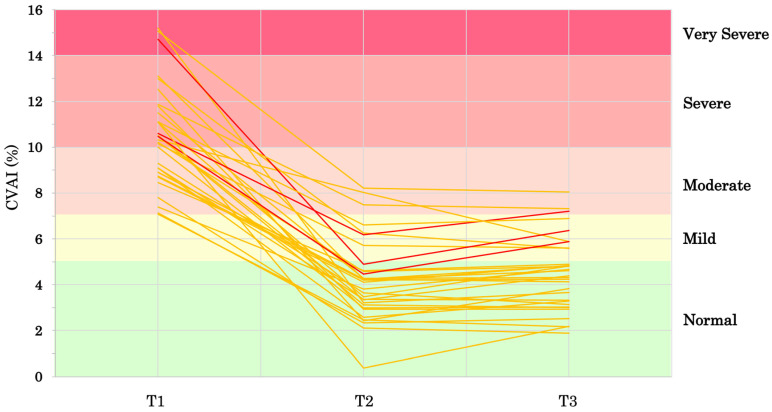
Individual changes in CVAI (*n* = 32). The three cases where the severity category worsened after treatment are indicated in red. CVAI, Cranial Vault Asymmetry Index; T1, at the start of helmet therapy; T2, at the end of helmet therapy; T3, at the chronological age of 1 year.

Figure 2 shows the infants’ CVAIs from T1 to T3. At T1, the median age was 4 months (range: 2–7 months) and the mean CVAI was 10.5 ± 2.2%. At T2, the median age was 9 months (range: 5–11 months) and the mean CVAI was 4.2 ± 1.8%. The change in the CVAI (ΔCVAI) from T1 to T2, reflecting the effectiveness of helmet therapy, was 6.3 ± 2.3%. From T2 to T3, the ΔCVAI was 0.3 ± 0.8%. Figure 3 shows the individual changes in the CVAI.

Table 3 presents the results of Friedman’s test for CVAI changes between T1 and T2 and between T2 and T3. A significant improvement was observed between T1 and T2 (*p* < 0.05), but there was no further change in the CVAI after helmet therapy between T2 and T3 (*p* = 0.26).

### 3.2. Study 2: Changes in Severity

Table 4 shows the distribution of severity between T1 and T2. The severity of plagiocephaly improved in all cases to moderate or below, with the majority of participants reaching the normal range. Table 5 shows the distribution of severity between T2 and T3. The severity improved in one infant (3%, from moderate to mild), showed no change in 28 infants (88%, 23 normal, 3 mild, 2 moderate), and worsened in three infants (9%, one from mild to moderate, two from normal to mild).

## 4. Discussion

The findings of the present study indicate that the severity of cranial deformity did not significantly worsen during the natural course from the end of helmet therapy to the chronological age of 1 year. Worsening severity was observed in 9% of the participants. There was minimal worsening in the CVAI measurement in several infants.

### 4.1. Infants’ Characteristics

Gender differences have been identified as a risk factor for DP [9,36,37,38,39,40]. In Japan, the birth sex ratio in the past years has been approximately 1.05 males for every 1 female [4]. However, among the subjects of this study, there were 2.2 times more males than females, which is consistent with previous reports.

Differences in physical characteristics at birth, such as height and weight, have been reported based on race and ethnicity [41]. Studies have shown that Japanese preschool children are smaller than those raised in Western countries and some other Asian countries, and they are also smaller than Japanese children raised in the United States [42]. The birth physical characteristics in this study, including the height, weight, and head circumference, were similar to the 50th percentile values reported in Japan [43]: height—48.1 cm, weight—2804 g (boys) and 2709 g (girls), and head circumference—33.0 cm. Additionally, updated data from the same year showed similar measurements: height—48.8 cm (boys) and 48.4 cm (girls); weight—3000 g (boys) and 2935 g (girls); head circumference—33.5 cm (boys) and 33.1 cm (girls); chest circumference—31.7 cm (boys) and 31.6 cm (girls) [42]. Although this study included two preterm infants, the participants were considered to reflect the typical physical characteristics of the general Japanese population.

In this study, it was not possible to determine whether vacuum deliveries were more frequent. However, delivery methods such as forceps and vacuum delivery are known risk factors for DP [36,38].

### 4.2. Changes in CVAI

In this study, the CVAI improved from 10.5 ± 2.2% at the start of helmet therapy to 4.2 ± 1.8% at the end of treatment. The helmet used in this study has been shown to be effective in improving moderate to severe DP [10]. The results indicate that the degree of improvement varies depending on the severity at the start of treatment, with a ΔCVAI improvement of −9.1% in the most severe cases, −6.6% in severe cases, and −4.4% in moderate cases. Other studies have also reported that the age at the start of helmet therapy and the severity of DP influence the effectiveness of treatment [6,8,14,21,26,44]. Our study’s results are consistent with these findings, suggesting that a significant level of treatment efficacy was achieved.

Furthermore, a change in CVAI of 0.3 ± 0.8% was observed between the end of helmet therapy and the chronological age of 1 year. Aarnivala H et al. reported that the head circumference grew at a similar rate between 3 and 6 months and between 6 and 12 months, but the rate of change in asymmetry-related variables was only 17–55% during the 6–12 month period, compared with that during the 3–6 month period [29]. In their study, multiple asymmetry-related variables were used, such as the Oblique Cranial Length Ratio, which measures diagonals on a two-dimensional plane; the Anterior Cranial Asymmetry Index and Posterior Cranial Asymmetry Index, which represent volumetric ratios; and the Asymmetry Score, Weighted Asymmetry Score, and Flatness Score, which were calculated based on 3D surface variables using the distribution of normal vectors. Although different methods were used in this study, the low rate of change observed in late infancy is consistent with the results of Aarnivala H et al. Additionally, Lo AL et al. found that the cranial growth rate was high between 1 and 2 months, but lower between 9 and 12 months [45]. As the cranial shape is influenced by external factors, the rate of change in late infancy is expected to be low, as seen in the present study’s results.

However, a previous study has reported that the head shape becomes rounder and more symmetrical between infancy and 18 months of age [46]. Previous studies have also shown that the greater the severity of cranial deformity, the more effective helmet therapy tends to be. If the degree of cranial deformity is severe when helmet therapy ends in late infancy, the change in ΔCVAI by the time the child reaches 1 year may be greater than the results observed in this study.

### 4.3. Changes in Severity

Regarding the change in severity, approximately 80% of infants with moderate or severe DP showed an improvement to within the normal range following helmet therapy, and significant changes in severity were observed. After helmet therapy, no significant changes in severity were noted by the time the infants reached 1 year of age.

Past studies tracking the natural course of cranial deformity from 1 to 6 months of age have shown that the severity of cranial deformity peaks at around 3 months, but, by 6 months, it improves to levels similar to those observed at 1 month of age [47]. Additionally, in children who exhibited severe cranial deformity between 4 and 8 months, about 70% remained severe even after approximately 3 months of natural development [13]. Our study’s findings, consistent with the findings of these reports, suggest that cranial deformity severity is more likely to change during the first half of infancy, with the changes becoming smaller as infants approach the later stages of infancy.

In this study, the three infants whose severity worsened were all full-term infants, and it was confirmed that they were able to crawl and stand with support by 1 year of age. One of these infants did not walk independently by 1 year and 5 months, but no significant developmental delays were observed. Due to the small sample size in this study, it was difficult to identify the specific causes of the worsening. However, by tracking the individual CVAI values (Figure 3), it was found that the greater the severity at the time of helmet therapy, the more likely there is to be a “V-shaped” change after treatment, with some cases worsening. Additionally, it is also possible that the earlier the treatment is completed, the more likely it is to result in a V-shaped change. However, in this study, one case where the severity improved by the age of 1 year had its treatment completed at 8 months of age. On the other hand, the three cases where the severity worsened had their treatment completed at 6 months, 7 months, and 9 months, respectively. Further studies with larger sample sizes are needed to identify which children are more likely to experience worsening and to explore the relationship between the age at treatment completion and post-treatment CVAI regression.

DP, especially positional plagiocephaly, is caused by external factors, so the primary preventive measure after helmet therapy, similar to early infancy, is ensuring that infants do not remain in a single position while sleeping. In the later stages of infancy, as motor development progresses, it is generally acceptable to allow the infant’s movements to dictate their positions. However, until the fontanelle closes, typically between 1 and 1.5 years of age, it is advisable for caregivers to remain conscious of the need for positional changes. Although the changes in cranial shape observed after treatment in this study were minimal, the results may help to alleviate concerns for parents after helmet therapy.

### 4.4. Changes Between Preterm and Term Infants

This study included two preterm infants. Launonen et al. confirmed that CA in preterm and full-term infants is nearly identical during the first 3 years of life [48]. It has also been shown that, once the corrected age exceeds 3 months, CA improves at the same rate in both preterm and full-term infants. Furthermore, previous studies have reported no significant difference in the incidence of plagiocephaly between preterm and full-term infants at chronological ages of 1, 3, and 6 months [49]. However, along with the age at the start of treatment and the initial asymmetry, prematurity is significantly correlated with the duration of head orthosis therapy [50]. Given the recognized differences in growth and development between preterm and full-term infants after birth, further investigation is needed to explore how these differences affect cranial shape changes.

### 4.5. Limitations

This study has some limitations. One limitation is the lack of a control group that did not receive helmet therapy. Moreover, we classified the patients solely based on the CVAI values and did not exclude infants with an abnormally high or low cephalic index. Therefore, there may have been differences in the CVAI changes between infants treated only for asymmetry and those treated for both asymmetry and either brachycephaly or dolichocephaly. One past report was prospective and randomized and compared helmet therapy with a control group without helmet therapy, and it followed the participants for up to 24 months of age, making it a very valuable report. Meanwhile, this study addresses not only plagiocephaly but also brachycephaly. However, the changes after helmet therapy up to 24 months of age over time were not clearly outlined [30]. Additionally, a comparison with this study was challenging due to the use of different indices for evaluation. Furthermore, a previous report has suggested that the treatment period for preterm infants might be shorter [50]; therefore, it is important to analyze full-term and preterm infants separately. Moreover, for preterm infants, it may be necessary to evaluate them based on their corrected age. In the future, considering these aspects, prospective studies with larger sample sizes are needed.

## 5. Conclusions

This study demonstrated that the severity of cranial deformity showed minimal worsening during the natural course from the end of helmet therapy to the chronological age of 1 year. This suggests that the results could alleviate parents’ concerns after helmet therapy. However, since DP is caused by prolonged periods of lying in the same position, it is important to continue encouraging the infant’s movement, even after completing helmet therapy.

## Figures and Tables

**Table 1:** Infants’ clinical characteristics (*n* = 32).

Male	22 (68.8)
Gestational age at birth, weeks	38.5 (34–41)
Birth height, centimeters	49 (45.5–52.2)
Birth weight, grams	2906 (2056–3828)
Birth head circumference, centimeters	33.8 (30.5–36)
Birth chest measurement, centimeters	31.7 (28.5–36.5)
Multiple birth	0 (0)
Mode of delivery:	
Vaginal	18 (56.3)
Vacuum	5 (15.6)
Caesarian	9 (28.1)
Intrauterine position:	
Cephalic	30 (93.8)
Breech	2 (6.3)

Data for gestational age at birth and birth measurements are shown as medians (min–max). Others are shown as numbers (percentage).

**Table 2:** Mothers’ clinical characteristics (*n* = 32).

Mother’s Age at Birth, Years	35 (25–43)
Primipara	17 (53.1)

Data for mother’s age at birth are shown as medians (min–max). The other data are shown as numbers (percentage).

**Table 3:** Changes in CVAI.

	T1	T2	T3	*p*–Value		
				Whole	T1–T2	T2–T3
CVAI, %	10.5 ± 2.2	4.2 ± 1.8	4.5 ± 1.6	<0.05	<0.05	0.26

The Cranial Vault Asymmetry Index is presented as the mean ± standard deviation. CVAI, Cranial Vault Asymmetry Index; T1, at the start of helmet therapy; T2, at the end of helmet therapy; T3, at the chronological age of 1 year.

**Table 4:** Distribution of severity between periods before and after helmet therapy.

				After Therapy			
		Normal	Mild	Moderate	Severe	Very Severe	Total
	Normal	0	0	0	0	0	0
	Mild	0	0	0	0	0	0
Before	Moderate	12	0	0	0	0	12
therapy	Severe	11	4	2	0	0	17
	Very severe	2	0	1	0	0	3
	Total	25	4	3	0	0	32

Data are shown as numbers.

**Table 5:** Distribution of severity between periods after helmet therapy and at the chronological age of 1 year.

			At the Chronological Age of 1 Year				
		Normal	Mild	Moderate	Severe	Very Severe	Total
	Normal	23	2	0	0	0	25
	Mild	0	3	1	0	0	4
After	Moderate	0	1	2	0	0	3
therapy	Severe	0	0	0	0	0	0
	Very severe	0	0	0	0	0	0
	Total	23	6	3	0	0	32

Data are shown as numbers.

## Data Availability

The data presented in this study are available on request from the corresponding author.
